# Investigation on the Effects of Modifying Genes on the Spinal Muscular Atrophy Phenotype

**DOI:** 10.1055/s-0042-1751302

**Published:** 2022-09-05

**Authors:** Drenushe Zhuri, Hakan Gurkan, Damla Eker, Yasemin Karal, Sinem Yalcintepe, Engin Atli, Selma Demir, Emine Ikbal Atli

**Affiliations:** 1 Department of Medical Genetics, Trakya University Faculty of Medicine, Edirne, Turkey; 2 Department of Pediatric Neurology, Trakya University Faculty of Medicine, Edirne, Turkey

**Keywords:** spinal muscular atrophy, modifying genes, neuromuscular disorder, *SMN1*, *SMN2*

## Abstract

**Introduction** Spinal muscular atrophy (SMA) is an autosomal recessive neuromuscular disorder caused by the degeneration of motor neurons, muscle weakness, and atrophy that leads to infant's death. The duplication of exon 7/8 in the *SMN2* gene reduces the clinical severity of disease, and it is defined as modifying effect. In this study, we aim to investigate the expression of modifying genes related to the prognosis of SMA like *PLS3* , *PFN2* , *ZPR1* , *CORO1C* , *GTF2H2* , *NRN1* , *SERF1A* , *NCALD* , *NAIP* , and *TIA1.*

**Methods** Seventeen patients, who came to Trakya University, Faculty of Medicine, Medical Genetics Department, with a preliminary diagnosis of SMA disease, and eight healthy controls were included in this study after multiplex ligation-dependent probe amplification analysis. Gene expression levels were determined by real-time reverse transcription polymerase chain reaction and delta–delta CT method by the isolation of RNA from peripheral blood of patients and controls.

**Results** *SERF1A* and *NAIP* genes compared between A group and B + C + D groups, and A group of healthy controls, showed statistically significant differences ( *p* = 0.037, *p* = 0.001).

**Discussion** *PLS3, NAIP* , and *NRN1* gene expressions related to SMA disease have been reported before in the literature. In our study, the expression levels of *SERF1A* , *GTF2H2* , *NCALD* , *ZPR1* , *TIA1* , *PFN2* , and *CORO1C* genes have been studied for the first time in SMA patients.

## Introduction

Spinal muscular atrophy (SMA) was first described by Guido Werdnig in 1891 in two baby siblings. Seven SMA cases were later reported by Johan Hoffman between 1893 and 1900. 1 SMA is a neuromuscular disease with an autosomal recessive inheritance that leads to early death with muscle weakness and atrophy, and it has a prevalence of 1 in 5,000 to 1 in 10,000. The SMA-associated chromosomal location is mapped in the 5q11.2–5q13.3 region. In 1994, the association between the survival motor neuron 1 ( *SMN1* ) gene and SMA was reported in the 5q13.2 region after defining the exact chromosomal location of the gene. 2 Exon 7/8homozygous deletion in the *SMN1* gene was detected in 95 to 98% of SMA patients, and point mutations in this gene were detected in 2 to 5% of patients. 3 Exon 7/8 homozygous deletion in the *SMN1* gene is the main factor in the diagnosis of SMA. The *SMN1* gene encodes the survival motor neuron (SMN) protein. As a result of a pathogenic variation, a defective or low-level expression of the SMN protein leads to the loss of function of α neuronal cells in the anterior horn region of the spinal cord, causing the skeletal muscles to weaken and shrink. It has been reported that the c.859G > C (p. Gly287Arg) variation in the seventh exon of the *SMN2* gene increases the copy number of the transcript at the mRNA level. The increase in the copy number of the *SMN2* gene contributes to the progression of the disease, and this has been defined as a modifying effect. 1 2 3 4 The *SMN1* and *SMN2* genes are mapped in the centromeric and telomeric parts of the chromosome 5q13.2 region. The nucleotide sequences between these two genes are more than 99% similar, encoding a 294-amino acid-long, 28-kDa SMN protein. 5 Although the SMN protein produced by the *SMN2* gene is sufficient for all cells, it is not enough for motor neurons. 6 As a result of the studies performed with heterogeneous nuclear ribonucleoproteins (hnRNPs), the SMN protein is determined to interact with the regulator gene of the glucosyltransferase (RGG) box. After the SMN protein forms a complex with the hnRNPs and the RGG box, the complex interacts with pre-mRNA and nuclear mRNA and plays an important role in the processing and transport of mRNA. 7 Owing to the faulty production of the SMN protein, snRNPs cannot interact with other molecules and the functions of motor axons are negatively affected because of splicing errors. 8 The faulty production of the SMN protein leads to defective functions of both molecular biological features and metabolic activities. 9 The clinical severity of SMA disease may differ among patients. Whereas some patients die in infancy, some with the same mutation may survive with many symptoms, such as muscle discomfort, wheelchair dependency, or milder clinical symptoms. Exon 7/8 homozygous deletion in the *SMN1* gene may cause different phenotypes in patients. Therefore, SMA is divided into five clinical subtypes according to physical examination findings: SMA type 0, type I, type II, type III, and type IV. 10 An increase in the number copies of the *SMN2* gene increases the clinical severity of SMA. 1 The results of the studies conducted in recent years have revealed the existence of new modifying genes in SMA and the *SMN2* gene. We know SMA is a monogenic disease but shows clinical heterogeneity. The severity of SMA clinic is related to *SMN2* , and this disease manifestation must have more modifying effects. So, the potential modifying genes were classified according to their functions in multiple protein interactions with SMN protein or relation to motor neuron survival, the effect on the promoter region, transcription, splicing, and expression parts. 11

This study aimed to investigate the association between the expression levels of genes considered to have modifying effects on SMA and the prognosis of the disease in patients with SMA.

The differences in the gene expression levels of *plastin 3 (PLS3), profilin 2 (PFN2), zinc finger (ZPR1), coronin 1C (CORO1C), general transcription factor 2H, polypeptide 2 (GTF2H2), neuritin 1 (NRN1), small EDRK-rich factor 1A (SERF1A), neurocalcin delta (NCALD), neuronal apoptosis inhibitory protein (NAİP)* , and *cytotoxic granule-associated RNA-binding protein (TIA1)* genes predicted to have modifying effects on SMA were evaluated statistically in the patient and control groups.

## Materials and Methods

### Patients and Control Subjects

Seventeen patients (5 female and 12 male) who applied to the outpatient clinic of Trakya University Hospital, Medical Genetics Department, Genetic Diseases Diagnosis Center with a prediagnosis of SMA were included in the study. Six patients had SMA type I, eight had SMA type II, and three had SMA type III according to the clinical prediagnosis. The patients were aged between 9 months and 15 years, and the ages of the individuals included in the control group were determined to be close to the average age of the patients. The control group was composed of eight individuals who did not have a neurological disease or had no family history. Among the SMA patients, deletions and duplications in the *SMN1* and *SMN2* genes were evaluated using multiplex ligation-dependent probe amplification (MLPA) probemix (P460-A1 and P060-B2, MRC-Holland Salsa) SMA carrier probes through the MLPA method. Patients with exon 7/8 homozygous deletion in the *SMN1* gene were included in the study. Table 1 presents the detailed patient information.

**Table 1:** Demographic characteristics of the patients, SMA types, family information, and MLPA results

	SMA type	Age	Gender	*SMN1*	*SMN2*	Relative marriage	Mother	Father
P1	Type II	14 y	M	Exon 7–8 homozygous deletion	Exon 7 (1.5-fold) increased	–	*SMN1* –7–8 Heterozygous deletion *SMN2-* Normal	*SMN1* –7–8 Heterozygous deletion *SMN2* –7 (1.5-fold) increased
P2	Type I	4 y	M	Exon 7–8 homozygous deletion	Normal	–	–	–
P3	Type II	9 y	M	Exon 7–8 homozygous deletion	Exon 7 (1.5-fold) increased	–	*SMN1* –7–8 Heterozygous deletion *SMN2-* Normal	*SMN1* –7–8 Heterozygous deletion *SMN2* –7 (1.5-fold) increased
P4	Type II	15 y	M	Exon 7 homozygous deletion Exon 8 heterozygous deletion	Exon 7 (1.5-fold) increased	–	–	–
P5	Type III	6 y	F	Exon 7–8 homozygous deletion	Exon 7 (1.5-fold) increased	–	–	–
P6	Type II	9 y	M	Exon 7 homozygous deletion Exon 8 heterozygous deletion	Exon 7 (1.5-fold) increased	–	*SMN1* –7–8 Heterozygous deletion *SMN2-* Normal	–
P7	Type I	11 y	M	Exon 7–8 homozygous deletion	Normal	–	–	–
P8	Type II	15 y	M	Exon 7–8 homozygous deletion	Exon 7 (1.5 fold) increased	–	S *MN1* –7–8 Heterozygous deletion *SMN2 -* 7. Heterozygous deletion	*SMN1-* 7–8 Heterozygous deletion *SMN2-* 7 (1.5-fold) increased
P9	Type I	3 y	M	Exon 7–8 homozygous deletion	Normal	–	–	–
P10	Type II	2 y	F	Exon 7–8 homozygous deletion	Normal	Yes (uncle's children)	–	–
P11	Type I	2 y	F	Exon 7–8 homozygous deletion	Normal	–	*SMN1* –7–8 Heterozygous deletion *SMN2-* Normal	*SMN1* –7–8 Heterozygous deletion *SMN2-* Normal
P12	Type III	3 y	F	Exon 7–8 homozygous deletion	Normal	–	–	–
P13	Type II	5 y	M	Exon 7–8 homozygous deletion	Exon 7–8 (1.5-fold) increased	–	–	–
P14	Type II	2 y	M	Exon 7–8 homozygous deletion	Exon 7–8 (1.5-fold) increased	–	*SMN1* –7–8 Heterozygous deletion *SMN2-* Normal	*SMN1* –7–8 Heterozygous deletion *SMN2-* Normal
P15	Type III	15 y	M	Exon 7–8 homozygous deletion	Exon 7–8 (1.5-fold) increased	–	–	–
P16	Type I	9 mo	F	Exon 7–8 homozygous deletion	Normal	–	*SMN1* –7–8 Heterozygous deletion *SMN2* –7–8 Homozygous deletion	*SMN1* –7–8 Heterozygous deletion *SMN2* –7 (1.5-fold) increased
P17	Type I	10 mo	M	Exon 7–8 homozygous deletion	Normal	Yes (aunt and uncle children)	*SMN1* –7–8 Heterozygous deletion *SMN2* -Normal	*SMN1* –7–8 Heterozygous deletion *SMN2* –7. Heterozygous deletion

Abbreviations: F, female; M, male; MLPA, multiplex ligation-dependent probe amplification; SMA, spinal muscular atrophy.

The patients signed an informed consent form prior to participating in the study. Ethics approval was granted by the Scientific Research Ethics Committee of Trakya University Faculty of Medicine (No. 16/30 dated January 10, 2018).

### Methods

A total of 2-mL peripheral venous blood taken from the patients and healthy controls was placed into ethylenediaminetetraacetic acid for RNA isolation, which was performed in accordance with the protocol of the kit (QIAGEN QIAamp RNA Isolation Kit, Germany). The concentration and purity (260/280 nm) of the isolated RNA samples were measured using a nanodrop device. RNA samples with the proper concentration and purity were stored at −80°C. Each RNA sample was transformed into complementary DNA (cDNA) in accordance with the protocol of the kit (Thermo Fisher Scientific High Capacity cDNA Reverse Transcription Kit, Lithuania). Subsequently, *PLS3* , *PFN2* , *ZPR1* , *CORO1C* , *GTF2H2* , *NRN1* , *SERF1A-H4F5* , *NCALD* , *NAIP* , and *TIA1* gene expression studies were performed by the TaqMAN Gene Expression Assay Kit (Thermo Fisher Scientific, Wilmington, MA, United States) in Applied Biosystems Step One Plus (Thermo Fisher Scientific, Wilmington, MA, United States) using appropriate primers and assays created specifically for each gene. The procedure was repeated three times for each patient on a 96-well plate. As an endogenous control, the β-actin housekeeping gene was used as an internal control.

The delta CT (ΔCt) values were calculated from the data obtained using the following formula: ΔCt = gene Ct-housekeeping Ct. 12 The values were calculated using ΔΔCt and 2-∆∆Ct, and the gene expression levels were determined.

## Results

Patients were divided into four groups based on the MLPA results:

*Group A:* This group comprised eight patients with *SMN1* exon 7/8 homozygous gene deletion and normal *SMN2* copy number (no increase in copy number) (P2, P7, P9, P10, P11, P12, P16, and P17). The most common clinical findings in patients were unable to sit without support, tongue fasciculation, decreased or absent reflexes, muscle weakness and atrophy, and hypotonia.
*Group B:* This group comprised four patients with *SMN1* exon 7/8 homozygous gene deletion and an increased copy number of exon 7 in the *SMN2* gene (P1, P3, P5, and P8). The patients could sit unsupported but never ambulated, and they had problems while walking or could not walk.
*Group C:* This group comprised two patients with *SMN1* exon 7 homozygous deletion/exon 8 heterozygous deletion and an increased copy number of *SMN2* exon 7 (P4 and P6). Patients had muscle weakness, they could sit by themselves, and they had late muscle development.
*Group D:* This group comprised three patients with *SMN1* exon 7/8 homozygous deletion and an increase in the copy number of *SMN2* exon 7/8 (P13, P14, and P15). Patients had problems while walking and muscle soreness.

The patients included in this study were divided into three clinical subtypes based on the clinical classification criteria of the 1991 SMA International Consortium:

*SMA type I:* Six patients (P2, P7, P9, P11, P16, and P17)
*SMA type II:* Eight patients (P1, P3, P4, P6, P8, P10, P14, and P15)
*SMA type III:* Three patients (P5, P12, and P13)

### 2 ^ (- ΔΔCt) Values of the Patient and Control Groups

For the expression levels of the relevant genes in the patient and control groups, each sample was analyzed three times based on the Ct values obtained at the end of each analysis. The expression of each gene was calculated as 2 ^ ^(- ΔΔCt)^ ( Table 2 ).

**Table 2:** Gene expression values of patients

	*PLS3*	*PFN2*	*ZPR1*	*CORO1C*	*GTF2H2*	*NRN1*	*SERF1A*	*NCALD*	*NAIP*	*TIA1*
P1	1.19	2.96	0.83	1.67	1.09	2.78	1.18	1.18	2.32	2.29
P2	1.48	0.14	0.05	0.78	0.10	0.41	0.52	0.21	0	0.46
P3	1.23	1.21	0.63	0.52	0.33	1.19	0.67	0.45	0.32	1.10
P4	1.22	1.79	0.41	1.33	0.45	0.36	0.41	0.32	0.46	0.70
P5	1.69	1.31	1.01	0.95	0.78	1.09	0.95	0.68	0.16	1.11
P6	0.93	0.55	0.43	0.65	0.44	2.78	0.65	0.34	0.93	1.06
P7	1.11	1.49	0.56	1.19	0.57	1.56	0.47	0.77	0.13	0.73
P8	1.44	2.17	1.16	1.44	0.63	1.73	0.77	0.61	0.63	0.77
P9	1.51	0.73	0.69	1.21	0.63	0.67	0.21	0.19	0.05	0.41
P10	2.83	0.87	1.43	1.03	0.55	4.29	0.56	0.60	0	0.95
P11	1.70	1.22	1.68	0.93	0.89	0.83	0.69	0.87	0.14	0.89
P12	9.08	0.92	1.61	1.39	0.65	1.11	0.63	0.97	0.08	1.28
P13	1.98	1.11	0.79	0.49	0.47	0.50	0.75	0.48	1.15	0.56
P14	2.96	0.84	1.56	0.83	0.57	3.39	0.81	0.46	0.18	1.56
P15	0.82	1.11	0.61	1.40	0.67	0.24	0.75	0.35	1.63	0.88
P16	2.46	0.90	0.92	1.34	0.62	1.29	0.59	0.72	0.18	1.67
P17	2.46	2.00	2.02	0.76	0.93	1.31	0.73	0.798	0.20	1.59

The Ct value of the *NAIP* gene could not be calculated in two patients (P2 and P10). This was interpreted as a deletion of the *NAIP* gene, and RNA primers were designed for confirmation. The expression of each gene in the control group was calculated as 2 ^ ^(- ΔΔCt)^ based on the Ct values obtained at the end of each study. Statistical analysis was performed ( Table 3 ).

**Table 3:** Gene expression values of controls

	*PLS3*	*PFN2*	*ZPR1*	*CORO1C*	*GTF2H2*	*NRN1*	*SERF1A*	*NCALD*	*NAIP*	*TIA1*
C1	2.27	1.89	0.85	0.54	0.42	0.33	1.03	1.13	0.29	0.94
C2	1.33	0.84	4.28	0.85	0.26	0.94	0.77	0.29	2.12	1.47
C3	2.96	2.01	1.04	0.61	0.65	0.45	0.99	0.63	0.43	0.84
C4	3.92	3.87	1.40	0.73	1.09	1.33	1.18	0.95	0.31	1.18
C5	2.14	0.80	1.00	0.64	0.40	0.48	0.70	0.49	1.11	0.99
C6	1.55	2.13	1.62	1.27	2.11	1.26	2.82	1.43	0.36	1.02
C7	2.65	1.46	1.14	0.86	0.53	1.79	1.58	0.69	0.74	1.15
C8	1.29	0.67	0.93	0.87	0.45	3.50	0.84	0.51	1.74	0.87

The results were presented as the mean ± standard deviation. The suitability of the quantitative data to normal distribution was determined using the Shapiro–Wilk test. Comparison of the gene expression levels *(PLS3* , *PFN2* , *ZPR1* , *CORO1C* , *GTF2H2* , *NRN1* , *SERF1A* , *NCALD* , *NAIP* , and *TIA1)* between the groups (control, group A and group B + C + D) and SMA types (type I, II, and III) was performed using the Kruskal–Wallis test.

The SPSS version 20.0 (License No: 10240642) program was used for statistical analysis by the Department of Biostatistics and Medical Informatics at Trakya University. A ( *p* ) value of less than 0.05 was considered statistically significant.

### Evaluation of Group A, group B + C + D, and the Control Group Using the Nonparametric Kruskal–Wallis Test

**Fig. 1:**
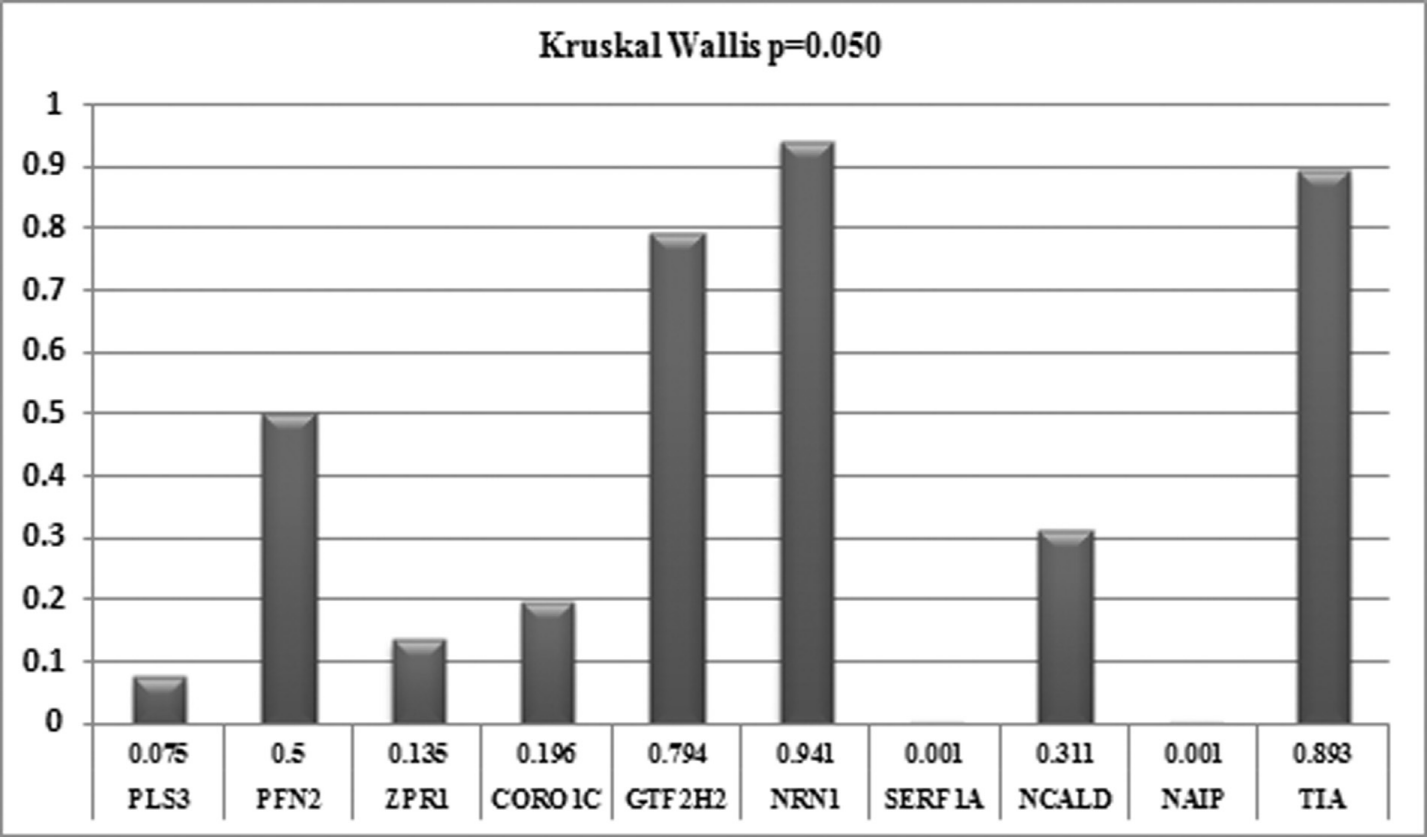
Nonparametric maintenance of A, B + C + D, and control groups.

A statistically significant difference was determined using the Kruskal–Wallis test in the *SERF1A* and *NAIP* gene expression levels among group A, group B + C + D, and the control group ( *p* = 0.001) ( Fig. 1 ).

#### SERF1A Gene Expression

**Fig. 2:**
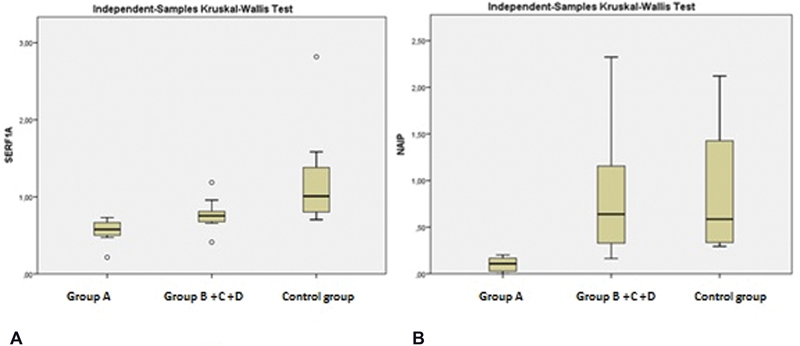
( **A** ) Comparison of SERF1A gene between groups. ( **B** ) Comparison of *NAIP* gene between groups.

A statistically significant difference was found in the *SERF1A* gene expression between group A and group (B + C + D) ( *p* = 0.037) and between group A and the control group ( *p* = 0.001). No statistically significant difference was observed in the *SERF1A* gene expression between group (B + C + D) and the control group ( *p* = 0.090) ( Fig. 2a ).

#### NAIP Expression

A statistically significant difference was found in the *NAIP* gene expression between group A and group (B + C + D) ( *p* = 0.001) and between group A and the control group ( *p* = 0.001). No statistically significant difference was observed in the *NAIP* gene expression between group (B + C + D) and the control group ( *p* = 0.873) ( Fig. 2b ).

#### P12

**Fig. 3:**
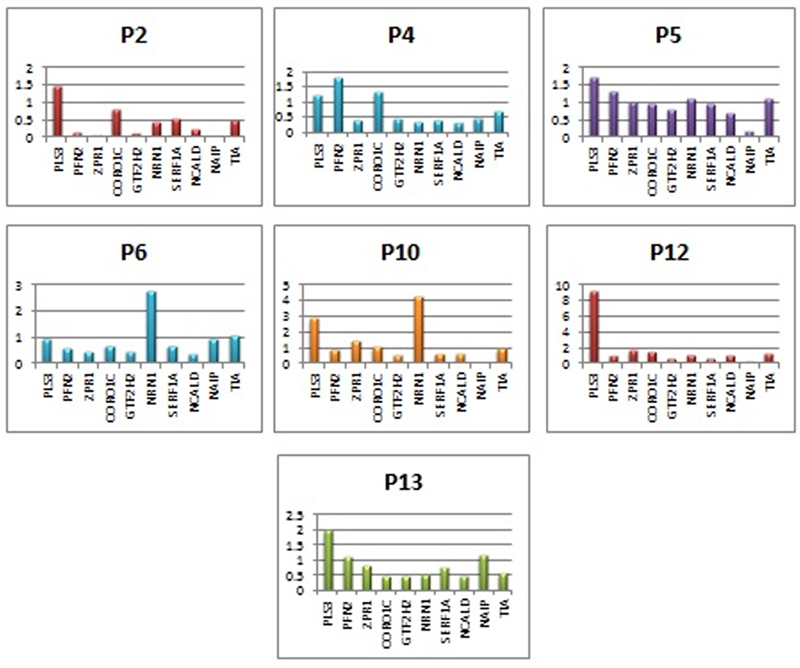
Expression level charts of P2, P4, P5, P6, P10, P12, and P13.

*SMA type III, group A* . We predicted that the patient with exon 7/8 homozygous deletion in the *SMN1* gene, normal *SMN2* gene copy number, and a clinical diagnosis of SMA type III clinical diagnosis was associated with a higher *PLS3* gene expression level (9.8 times) compared with other patients and the control group ( Fig. 3 ).

#### P10

**Fig. 4:**
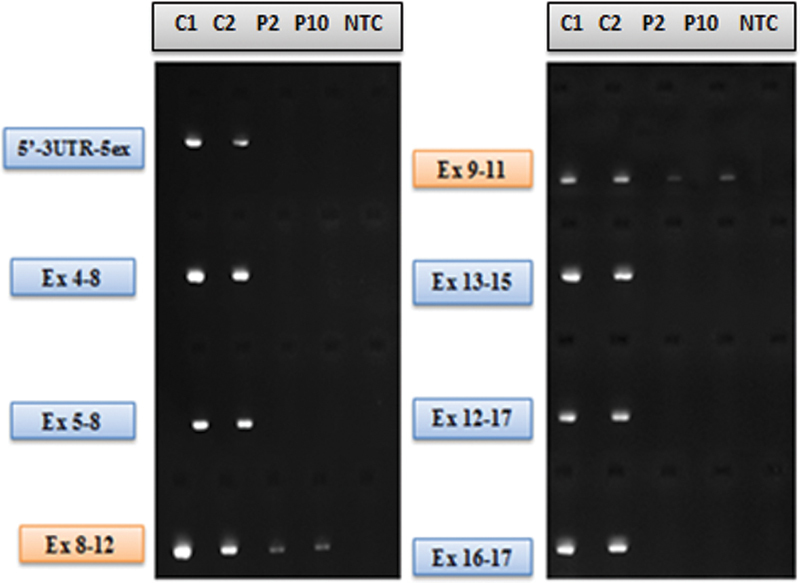
Agarose gel electrophoresis image of the *NAIP* gene (RNA primers designed specifically for the NAIP-208 ENST00000517649.6 transcript). The results of the real-time PCR analysis showed that the cDNA of control 1 (C1), control 2 (C2), P2 with an undetected Ct value and NC (negative control) were amplified by PCR to exclude possible contamination. Amplicons obtained by PCR were subjected to agarose gel electrophoresis and imaged in an ultraviolet transilluminator. No band pattern was seen in P2, P10, and NTC. This result excluded possible contamination while confirming the NAIP gene deletion at the same time. The nonspecific band patterns seen in exons 8 to 12 and exons 9 to 11 in P2 and P10 were interpreted as possible amplification of the NAIP pseudogene. PCR, polymerase chain reaction.

*SMA type II, group A* . We predicted that the patient with exon 7/8 homozygous deletion in the *SMN1* gene, normal *SMN2* gene copy number, and a clinical diagnosis of SMA type II was associated with a higher *NRN1* gene expression level (4.29 times) compared with other patients and the control group ( Fig. 3 ). The result of the real-time polymerase chain reaction (PCR) analysis showed that the Ct value associated with the *NAIP* gene expression level could not be detected in our patient. Anticipating that this could be related to a possible deletion in the *NAIP* gene, we designed an RNA primer specific to the *NAIP* gene. Exon regions with no band patterns were detected by PCR, and agarose gel electrophoresis confirmed the deletion ( Fig. 4 ).

#### P2

*SMA type I, group A* . The Ct value associated with the *NAIP* gene expression level was not detected ( Fig. 3 ) as a result of the real-time PCR analysis in the patient with exon 7/8 homozygous *SMN1* gene deletion and normal *SMN2* gene copy number. Anticipating that this could be related to a possible deletion in the *NAIP* gene, we designed an RNA primer specific to the *NAIP* gene. Exon regions with no band patterns were detected by PCR, and agarose gel electrophoresis confirmed the deletion ( Fig. 4 ).

#### P5

*SMA type III, group B* . The patient with exon 7/8 homozygous deletion in the *SMN1* gene and an increase in the *SMN2* exon 7 copy number had a clinical diagnosis of SMA type III, unlike the other patients in group B (P1, P3, and P8). Thus, this could be related to another gene expression level investigated previously in our study ( Fig. 3 ).

#### P4, P6

*SMA type II, group C* . In these two patients, we suggested that the heterozygous deletion in the exon 8 *SMN1* gene did not contribute to the progression of the patients' clinic, as P4 and P6 had the same clinical diagnosis (SMA type II) and P14 and P15, which are in group D, had exon 8 homozygous deletion in the *SMN1* gene ( Fig. 3 ).

#### P13

*SMA type III, group D* . The patient with exon 7/8 homozygous deletion in the *SMN1* gene and an increased copy number in exon 7/8 in the *SMN2* gene had an SMA type III clinical diagnosis, unlike the other patients in group D (P14, P15). Thus, this could be related to another gene expression level other than those investigated in our study ( Fig. 3 ).

**Fig. 5:**
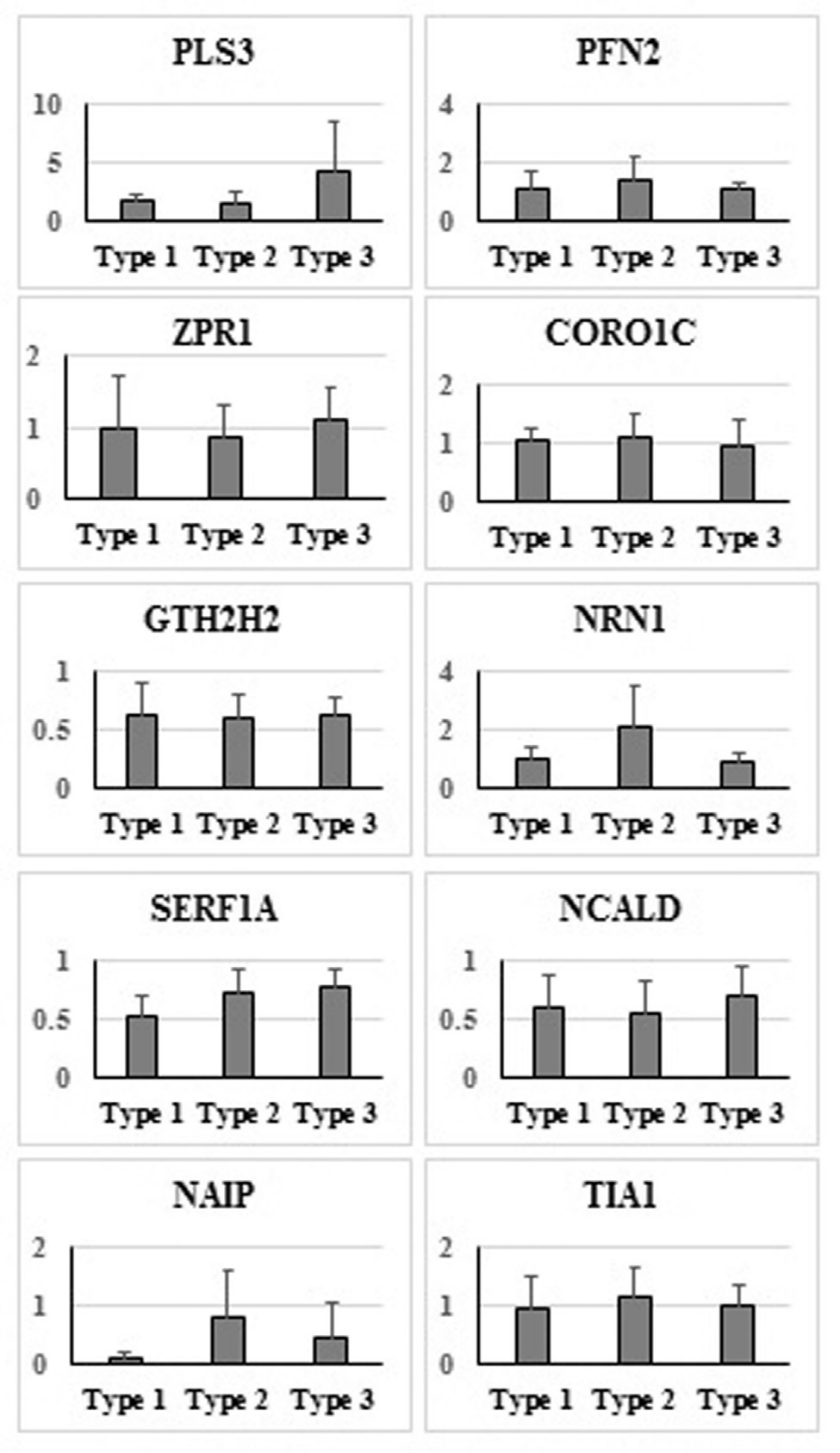
Comparison of expression levels according to SMA clinical types. SMA, spinal muscular atrophy.

The comparison of the expression levels according to SMA clinical type showed that the *PLS3* expression level increased in SMA type III and that the *NAIP* expression level decreased in SMA type I ( Fig. 5 ).

The mean *PLS3* gene expression level was found to be 1.8, 1.6, and 4.25 in SMA type I, type II, and type III, respectively. Although the *PLS3* gene expression level increased in SMA type III, no statistically significant difference was found between SMA type I and type II ( *p* = 0.197) ( Fig. 5 ). This result could be related to the low number of patients included in the SMA type III patient group (P5, P12, and P13). A statistically significant result could be obtained if the study were repeated with an increased number of patients.

The mean *NAIP* gene expression level was found to be 0.12, 0.81, and 0.46 in SMA type I, type II and type III, respectively. Although the *NAIP* gene expression level was lower in SMA type I than in types II and III, no statistically significant difference was found ( *p* = 0.081) ( Fig. 5 ). The low average *NAIP* expression level in SMA type I could be related to the failure to obtain the *NAIP* gene-specific Ct value in our two patients (P2 and P10). Statistically significant results could be obtained if the study were repeated with an increased number of patients.

## Discussion

SMA is an autosomal recessive neuromuscular disease characterized by weakness and atrophy in the proximal muscles. Homozygous deletion in the 7th and 8th exons of the *SMN1* gene or only in the seventh exon was found in 95% of the patients. As a result of the deletions in these exons, weakness and atrophy in skeletal muscles can be observed, as the SMN protein cannot be produced or is damaged. 13

Although the protein expressed by the *SMN2* gene compensates for the deficiency of the protein that the *SMN1* gene cannot express, some patients do not achieve the expected level of recovery. The better clinical course in SMA patients with an increased number of *SMN2* gene copies was interpreted as the modifying effect of the *SMN2* gene. 14

In some single-gene disorders, the effect of the mutation in a particular gene on the phenotype may differ among individuals carrying the same mutation. This can be explained by the change in expressivity and/or penetrance. Similar phenotypic differences can also be detected in SMA patients. The SMA consortium has clinically divided SMA into five subtypes, namely SMA type 0, SMA type I, SMA type II, SMA type III, and SMA type IV, taking into account the physical examination findings of the patients. 10

In his study on humans and mice, Nadeau 15 reported that modifying genes could be a factor affecting the clinical course of a disease (age and penetrance). The detection of modifying genes will contribute to a better understanding of the pathogenesis of SMA and to the development of drug studies. 14 Riordan et al also emphasized the importance of modifying genes in their studies. 16

In the present study, to explain the prognostic differences in patients diagnosed with SMA, we investigated the expression levels of *PLS3* , *PFN2* , *ZPR1* , *CORO1C* , *GTF2H2* , *NRN1* , *SERF1A* , *NCALD* , *NAIP* , and *TIA1* genes, which we predicted to have modifying effects on the patient and healthy control groups. We found a statistically significant difference in the *SERF1A* and *NAIP* gene expression levels ( *p* = 0.001). The *SERF1A* gene expression level being low and the prognosis being worse in the patients in group A could be explained by the modifying effect of the *SERF1A* gene. Considering the results on the *NAIP* gene expression level, we found that the NAIP gene also had a modifying effect.

Arkblad et al investigated the relationship between SMA disease and the *SERF1A* gene using the MLPA method. They reported that a deletion in an allele of the *SERF1A* gene was detected in all SMA type I patients included in the study, 50% of SMA type II patients, and 31% of SMA type III patients. However, no significant relationship was observed between the number of copies of the *SERF1A* gene and the clinical severity of SMA. 17

In their study performed on 26 SMA patients, Amara et al found that the copy number of the *SERF1A* gene was observed as one copy in exon 1 in 60% of the mild SMA type I cases and as two copies in the mild clinical ones of the SMA type II and type III cases. 18

Medrano et al reported that a deletion in the *NAIP* and *SERF1A* genes was observed in approximately 73 and 35% of SMA type I patients, respectively. As a result, deletions in the *NAIP* and *SERF1A* genes were detected in 90% of SMA type II and type III patients and 21% of SMA type I patients, as these two genes could have modifying effects. 19

In 34 SMA patients, Tran et al investigated the *SMN2* and *NAIP* genes, which are considered to have modifying effects. The *SMN2* gene copy number was detected as three copies in only one of the 13 patients with SMA type I and two copies in the remaining patients; three copies in 9 of the 11 patients with SMA type II and two copies in the remaining patients; and two copies in 2 of the 10 patients with SMA type III, three copies in five patients, and four copies in the remaining three patients. This result was reported as a modifying effect of the *SMN2* gene. The *NAIP* gene was observed as a homozygous deletion in five SMA patients, a single copy in 20 patients, and a normal copy number in nine patients. Therefore, it was interpreted as the modifying effect of the *NAIP* gene. 20

No studies have yet examined the relationship between SMA prognosis and the expression levels of *SERF1A* and *NAIP* genes. *SERF1A* gene function is a general regulator of protein aggregations, and *NAIP* gene function is related to the negative regulator of motor-neuron apoptosis. 21 According to their functions, *NAIP* gene is directly related to *IAP* (Inhibitor of Apoptosis) apoptosis family and has direct effect on motor neurons which are related to SMA prognosis, and *SERF1A* gene may regulate protein aggregation of *SMN* proteins; the location of *SERF1A* is near to *SMN1* and *SMN2* gene but there is not yet any functional study performed about the contribution of SMA prognosis for these two genes.

So, the expression levels of the *SERF1A* and *NAIP* genes being lower in SMA type I patients and the statistical comparisons between the groups in our study support the idea that the *SERF1A* and *NAIP* genes have modifying effects. 19 20

As a result of the comparison of the expression levels of the genes with SMA (SMA type I, SMA type II, and SMA type III) types, the *PLS3* gene expression increased in SMA type III and the *NAIP* gene expression decreased in SMA type I. However, we did not find a statistically significant difference between the SMA clinical types and the expression levels of the *PLS3* and *NAIP* genes. We considered this result to be related to the low number of patients included in our study. The result can become statistically significant if the number of patients is increased.

The *NRN1* gene synthesizes the protein required for the growth of neurite. In the only study in the literature examining the relationship between the SMA and the *NRN1* gene, the expression levels of the *PLS3* and *NRN1* genes were analyzed in nine patients with SMA diagnosis in four different families. Both siblings, P1 (21 months old, unable to walk) and P2 (14 years old, able to walk), in the first family were diagnosed with SMA type III, and the expression level of the *NRN1* gene was reported to be 0.9 and 1.4 times higher in P1 and P2, respectively. This result was interpreted as a modifying effect of the *NRN1* gene. No statistically significant difference was found between the *PLS3* gene expression level and the clinical course of SMA. In the second family, three siblings (P3, P4, and P5) with SMA type III diagnosis were included in the study, and the clinical course of H4 was more severe in P3 and P5. Contrary to the result for the first family, the *PLS3* gene had a modifying effect but the *NRN1* gene had no modifying effect. In the third family, two siblings were diagnosed with SMA type III, and the expression level of the *PLS3* gene was found to be associated with the clinical course. Moreover, the *NRN1* gene expression level was found to be unrelated to the clinical course in the third family, and the gene had no modifying effect. In the fourth family, two siblings were diagnosed with SMA type II (P9) and type III (P8) and included in the study. The *PLS3* gene expression level was determined to be 1.7 in the sibling diagnosed with SMA type II (P9) and 0.8 in the sibling diagnosed with SMA type III (P8). Thus, the *NRN1* gene was reported to have no modifying effect. 21 22

In this study, the *NRN1* gene expression was higher (4.29 times) in our patient with SMA type II (P10) than in the other patients and the healthy control group. This result supports the literature reporting the modifying effect of the *NRN1* gene.

In the study in which the *PLS3* gene expression and SMA relationship were investigated in 88 SMA patients (29 males less than 11 years old, 12 males older than 11 years old, 29 prepubertal females, and 18 postpubertal females), the highest *PLS3* gene expression was found in SMA type III postpubertal females. *PLS3* gene expression was reported as a modifier gene in females, as it varied according to age and puberty stage. 23

Analyzing the *PLS3* gene expression levels in 19 SMA type I patients, 21 SMA type II patients, 25 SMA type III patients, and 59 healthy controls, Yanyan et al evaluated the *SMN2* copy number using the MLPA method and found three copies of the *SMN2* gene in 76.9% of the patients, two copies in 21.5% of the patients and four copies in 1.5% of the patients. The *PLS3* gene expression levels were found to be 56.7% lower in SMA type II patients (i.e., those with one and two copies of the *SMN2* gene) than in SMA type III patients. The *PLS3* gene expression was 62.6% less in SMA type II patients (i.e., those with three copies of the *SMN2* gene) than in SMA type III patients. 24

This study showed that *PLS3* gene expression was increased 9.8 times in our SMA type III patient (P12 with SMN1 gene exon 7/8 homozygous deletion and *SMN2* gene exon 7/8 without an increased copy number) compared with other patients and the healthy controls. Although the *SMN2* copy number of our patient was normal, the good clinical course could be related to the increased *PLS3* gene expression. This result can be interpreted as the modifying effect of the *PLS3* gene.

He et al investigated the copy number changes in the *SMN2* , *NAIP* , *GTF2H2* , and *H4F5* genes in 157 SMA patients and found that the *SMN2* gene copy number was 8.72% single copy, 73.83% two copies, 15.43% three copies, and 2.01% four copies. They showed that all patients with a single *SMN2* gene copy number having a diagnosis of SMA type I support the findings about the modifying properties of the *SMN2* gene. In the same study, the *NAIP* gene copy number changes were evaluated in 149 patients and homozygous deletion was found in 15 patients, heterozygous deletion in 126 patients, and normal in eight patients. 25

Liu et al examined the *NAIP* and *GTF2H2* genes in 75 patients consisting of 41 SMA type I patients, 29 SMA type II patients, and five SMA type III patients. The *SMN2* gene was found as two copies in 28 patients, three copies in 29 patients and four copies in 18 patients. They reported that *NAIP* and *GTF2H2* gene deletions were detected in five patients (fourth exon in four patients and fifth exon deletion in one patient) and 10 patients, respectively. 26 In our study, we did not find any statistically significant results supporting the modifying effect of the *GTF2H2* gene expression level.

In their study conducted on mice, Torres-Benito et al found that antisense oligonucleotide therapy targeting the *NCALD* gene was effective for SMA disease. 27

No study investigating the *NCALD* gene expression level in SMA patients has been reported in the literature. Our study is the first to examine the relationship between SMA and the *NCALD* gene expression level. We found no statistically significant difference between the SMA disease phenotype and the *NCALD* gene expression level.

More than one study has examined the modifying effect of the *ZPR1* gene on SMA patients, and most of these studies were conducted by Gangwani et al. In their study on mice, Gangwani et al reported that the deficiency of the ZPR1 protein synthesized by the *ZPR1* gene caused neurodegeneration. 28

Ahmad et al analyzed the changes made by increasing and decreasing the expression level of the *ZPR1* gene in mice with SMA and found that the low expression level of the *ZPR1* gene caused loss of motor neurons, hypermyelination of the phrenic nerves, respiratory distress, and a more severe clinical course. They suggested that the high expression level of the *ZPR1* gene stimulates neurite growth and repairs axonal growth defects. 29 Genabai et al also reported that the *ZPR1* gene had a positive effect on *SMN2* gene expression. 30

Note that most of the studies that have investigated the relationship between the *ZPR1* gene and SMA are mice studies. Unlike the literature, our study is the first to analyze the relationship between the *ZPR1* gene expression level and SMA in humans. No statistically significant relationship was found between the *ZPR1* gene expression level and the SMA phenotype.

The *TIA1* gene regulates alternative splicing at the seventh exon of the SMN2 gene. Owing to this feature, it has been defined as a positive regulator in SMA disease. 31 In the literature, there is no study investigating the relationship among the *TIA1* gene copy number, the *TIA1* gene expression level, and SMA. In this respect, our study is the first to analyze the association between the *TIA1* gene expression level and SMA. No statistically significant difference was found between the *TIA1* gene expression level and the SMA phenotype in this study. 32

Wadman et al investigated the *PFN2* gene variations in SMA patients using the DNA sequence analysis method but could not find any significant relationship. 33 No statistically significant difference was found in our study, which is the first to examine the relationship between the *PFN2* gene expression level and SMA.

In their study on the functions of *PLS3* and *CORO1C* genes in SMA patients, McCabe et al 32 reported that *PLS3* and *CORO1C* genes could interact with F-actin and *SMN1* protein. 33 However, no study has yet investigated the expression level of the *CORO1C* gene in SMA disease. In this respect, our study is the first to analyze the relationship between the *CORO1C* gene expression level and SMA, and we found no statistically significant difference.

There are some limitations of this study. The number of patients in this study is low. Besides, this study does not include or exclude the effect of studied modifier genes depending on the treatments. Conducting similar studies in different populations with an increased number of patients can provide important insights into SMA disease and make significant contributions to the literature.

## Conclusion

The results of our study, in which we investigated the relationship between the expression levels of *PLS3* , *NAIP* , and *NRN1* genes and SMA, were previously reported in the literature. However, no study has yet investigated the relationship between the expression levels of *SERF1A* , *GTF2H2* , *NCALD* , *ZPR1* , *TIA1* , *PFN2* , and *CORO1C* genes and SMA. Therefore, this study is the first of its kind in the literature.

Although the results of the study support the modifying effects of *SERF1A* , *NAIP* , *NRN1* , and *PLS3* genes in SMA, we did not find a statistically significant difference in the modifying effects of *GTF2H2* , *NCALD* , *ZPR1* , *TIA1* , *PFN2* , and *CORO1C* genes on SMA.
